# An M cell-targeting recombinant *L. lactis* vaccine against four *H. pylori* adhesins

**DOI:** 10.1007/s00253-024-13070-0

**Published:** 2024-02-23

**Authors:** Furui Zhang, Tianyi Shi, Zhen Zhang, Shue Wang, Jing Liu, Yonghong Li, Xuequan Wang, Kunmei Liu, Le Guo

**Affiliations:** 1 School of Pharmacy, Ningxia Medical University, Yinchuan, 750004 China; 2 School of Laboratory, Ningxia Medical University, Yinchuan, 750004 China; 3 Department of Geriatrics and Special Needs Medicine, General Hospital of Ningxia Medical University, Yinchuan, 750004 China; 4 School of Public Health and Management, Ningxia Medical University, Yinchuan, 750004 China; 5 Key Laboratory of Radiation Oncology of Taizhou, Taizhou Hospital of Zhejiang Province Affiliated to Wenzhou Medical University, Taizhou, 317000 China; 6 Ningxia Key Laboratory of Cerebrocranial Diseases, Ningxia Medical University, Yinchuan, 750004 China; 7 Ningxia Key Laboratory of Clinical and Pathogenic Microbiology, General Hospital of Ningxia Medical University, Yinchuan, 750004 China

**Keywords:** M cell, Recombinant *L. lactis*, *H. pylori*, Vaccine surface display, Adhesin

## Abstract

**Abstract:**

The acidic environment and enzyme degradation lead to oral vaccines often having little immune effect. Therefore, it is an attractive strategy to study an effective and safe oral vaccine delivery system that can promote gastrointestinal mucosal immune responses and inhibit antigen degradation. Moreover, the antigens uptake by microfold cells (M cells) is the determining step in initiating efficient immune responses. Therefore, M cell-targeting is one promising approach for enhancing oral vaccine potency. In the present study, an M cell-targeting *L. lactis* surface display system (plSAM) was built to favor the multivalent epitope vaccine antigen (FAdE) to achieve effective gastrointestinal mucosal immunity against *Helicobacter pylori*. Therefore, a recombinant *Lactococcus lactic acid* vaccine (LL-plSAM-FAdE) was successfully prepared, and its immunological properties and protective efficacy were analyzed. The results showed that LL-plSAM-FAdE can secretively express the recombinant proteins SAM-FAdE and display the SAM-FAdE on the bacterial cell surface. More importantly, LL-plSAM-FAdE effectively promoted the phagocytosis and transport of vaccine antigen by M cells in the gastrointestinal tract of mice, and simulated high levels of cellular and humoral immune responses against four key *H. pylori* adhesins (Urease, CagL, HpaA, and Lpp20) in the gastrointestinal tract, thus enabling effective prevention of *H. pylori* infection and to some extent eliminating *H. pylori* already present in the gastrointestinal tract.

**Key points:**

• *M-cell-targeting L. lactis surface display system LL- plSAM was designed*

• *This system displays H. pylori vaccine-promoted phagocytosis and transport of M cell*

• *A promising vaccine candidate for controlling H. pylori infection was verified*

**Supplementary Information:**

The online version contains supplementary material available at 10.1007/s00253-024-13070-0.

## Introduction

*Helicobacter pylori* (*H. pylori*) become the main reason for chronic gastritis, peptic ulcer, stomach cancer, and gastric mucosa-associated lymphoid tissue (MALT) lymphoma (Czinn and Blanchard 2011). In view of the undesirable effect of antibiotic-based therapies, efficient prophylactic or therapeutic vaccines against *H. pylori* are considered the best public health measure (Sutton and Boag 2019). However, there is still no effective and safe *H. pylori* vaccine available for humans. Adhesins promote *H. pylori* binding to host cell receptors, which results in persistent colonization and infection (Kao et al. 2016). In our previous studies, we designed an epitope vaccine CFAdE comprising cholera toxin B subunit (CTB) and poly-epitope peptide FAdE constructed by four *H. pylori* adhesins (urease, Lpp20, HpaA, and CagL). And the CFAdE vaccine provided a protective effect on *H. pylori* infection (Guo et al. 2017b). CFAdE is an oral vaccine, but it currently lacks a potent and reliable vaccine delivery mechanism that guards against stomach acid damage and promotes a robust and long-lasting mucosal immune response.

Several investigations have demonstrated that *Lactococcus lactis*, a *lactic acid bacterium* (LAB), has the capacity to serve as a mucosal vaccine delivery vehicle (Batista et al. 2020). The fact that *Lactobacillus* is generally regarded as safe (GRAS) has been demonstrated by centuries of usage in food fermentation (Bourdichon et al. 2012). Moreover, *L. lactis* can boost the immune response as an adjuvant by virtue of its immunomodulatory properties and probiotic functions (Wang et al. 2016). So far, several bacterial and viral antigens, including HIV (Gram et al. 2007), hepatitis B virus (Zhang et al. 2011), and *H. pylori* (Aliramaei et al. 2019), have been effectively produced in *L. lactis*. Moreover, there is no need for further purification of antigen components when *L. lactis* is chosen as a vaccine delivery vehicle. The surface of *L. lactis* as an antigen display system is more consistent with the design requirements of mucosal vaccines (Raha et al. 2005). So, *L. lactis* surface display for mucosal vaccine has become the focus of many scientists’ research.

M cells located in the follicle-associated epithelium of intestinal Peyer’s patches serve as antigen-collecting portals (Kimura 2018). M cell uptake and transport of antigens is a key first step in initiating systemic and mucosal response (Rios et al. 2016). And thus, oral vaccine development based on M cell-targeting has a more far-reaching prospect. In this study, the M cell-targeting *L. lactis* surface display system (plSAM) was constructed to assist FAdE (poly-epitope peptide of urease, CagL, HpaA, and Lpp20) in obtaining effective gastrointestinal mucosal immunity against *H. pylori*. Eventually, a recombinant vaccine LL-plSAM-FAdE was prepared and its protective efficacy and immunological properties against *H. pylori* infection were analyzed.

## Materials and methods

### Design M cell-targeting *L. lactis* surface display system plSAM

SPusp45 is an N-terminal 22 amino acid signal peptide from the secreted extracellular protein USP45 of *L. lactis*. It has been discovered that PS, a brief synthetic propeptide, improves *L. lactis*’s heterologous secretion efficiency. Mtp is a designed M cell-targeting peptide based on Cpe17, Co1, and CKS-9 cell-targeting ligands. AcmA is an autolysin of *L. lactis* (Steen et al. 2005). The C-terminal (cA) region of AcmA is involved in bacterial cell wall binding (Steen et al. 2005). To obtain the plasmid plSAM, the core component SAM consisting SPusp45, PS, cA, multiple clone site (MCS), and Mtp is synthesized. The plasmid plSAM was constructed by SAM fragments inserting into the plasmid pNZ8148 by *Nco* I and *Hin*d III.

### Design recombinant *L. lactis* LL-plSAM-FAdE

The FAdE gene was amplified from the pCzn1-CFAdE plasmid and inserted into plSAM using *Kpn* I and *Xba* I to produce the expression vector plSAM-FAdE. Finally, the recombinant *L. lactis* LL-plSAM-FAdE was built after expression vector plSAM-FAdE were transformed into *L. lactis* NZ9000 successfully*.* The whole nucleotide sequence and amino acid sequences of plSAM-FAdE are provided in Table S1.

### Expression and analysis of LL-plSAM-FAdE

LL-plSAM-FAdE was cultivated in M17 medium containing chloramphenicol (Cm, 10 μg/mL) at 30 ℃. The SAM-FAdE expression from LL-plSAM-FAdE was inducted by 1 ng/mL nisin (Guo et al. 2022). The production of SAM-FAdE protein by recombinant *L. lactis* LL-plSAM-FAdE was analyzed by SDS-PAGE, Western blot, and immunofluorescence staining. Centrifugation at 12,000 g for 10 min was performed to harvest the cultured cells. Bacterial pellets were lysed and centrifuged for l5 min at 15,000 g. The precipitation and supernatant extracts were subjected to SDS-PAGE and then transferred to a polyvinylidene fluoride (PVDF) membrane for 1 h at 100 V. The PVDF membrane was blocked with 5% (w/v) skimmed milk and incubated with mouse anti-FAdE polyclonal antibody at dilution of 1:1000, and was then treated with goat anti-mouse IgG (1:2000). For immunofluorescence staining, the LL-plSAM-FAdE or LL-plSAM was washed and harvested. The mouse anti-FAdE polyclonal antibody at dilution of 1:1000 was added and stained for 12 h, followed FITC-labeled goat anti-mouse IgG stain for 1 h. The samples were visualized using a fluorescence microscope.

### Whole-cell ELISA

The LL-plSAM-FAdE or LL-plSAM was resuspended in PBS. ELISA plates were percoated with the SAM-FAdE protein (0.5 μg/well) and different amounts of bacteria. After three repeated washes, the plates were blocked with 0.5% BSA. The SAM-FAdE proteins were incubated with mouse anti-FAdE antiserum (1:1000) for 1 h, followed incubation with HRP-labeled goat anti-mouse IgG. Then, 100 μL tetramethylbenzidine was added into each well of the plates. After 10 min at room temperature, 50 μL 2 M H_2_SO_4_ was added into each well to stop the reaction. The absorbance was read at 450 nm.

### Preparation and analysis of antibodies specific for LL-plSAM-FAdE

All animal experiments were approved by the Animal Ethical and Experimental Committee of Ningxia Medical University. Specific pathogen-free (SPF) BALB/c mice (male, 5–6 weeks old; *n* = 6) were immunized orally with 3 × 10^9^ CFU of LL-plSAM-FAdE on days 1, 2, 8, 9, 15, 16, 22, and 23. Seven days after the last oral immunization, the antiserum was prepared as previous reports (Guo et al. 2022). The specificity of antiserum was analyzed by ELISA and Western blot. For ELISA analysis, the plates were coated with 0.5 μg/well of SAM-FAdE, urease, CagL, HpaA, Lpp20, or BSA. The LL-plSAM-FAdE antiserum was diluted 1:500. For Western blot analysis, *H. pylori* antigens (urease, UreA, UreB, CagL, HpaA, and Lpp20) were electroblotted onto PVDF membrane. The PVDF membrane was incubated with LL-plSAM-FAdE antiserum at dilution of 1:1000 and was then treated with HRP-labeled goat anti-mouse IgG.

### *H. pylori* urease neutralization assay

Urease is one of the key factors that *H. pylori* can survive under acidic stomach conditions. A urease neutralization assay was used to identify whether the LL-plSAM-FAdE vaccine could stimulate the production of neutralizing antibodies that effectively inhibit *H. pylori* urease activity (Zhou et al. 2009). Briefly, *H. pylori* urease (2 µg in 50 µL) added with different concentrations of antiserum was incubated at 4 ℃ overnight. Then, 100 µL of urea phenol red solution containing 0.02% phenol red and 500 mM urea was added. The color changes were detected at 550 nm for 3 h with a time interval of 30 min. The percentage inhibition of urease activity was calculated as previously reported (Zhou et al. 2009).

### Adherence inhibition assay

Adhesion inhibition assay was used to analyze the inhibitory effect of antiserum on *H. pylori* adhesion to gastric mucosal cells (Zhou et al. 2009). *H. pylori* suspension (1 × 10^9^ CFUs/mL) was incubated with different antiserum concentrations for 1 h. Then, the above-treated *H. pylori* was added to gastric mucosal cells GES-1 and co-incubated for 2 h at 37 ℃. After Giemsa staining, *H. pylori* adhesion ability to GES-1 cells was visualized and calculated by oil immersion microscopy.

### M-cell targeting property analysis

The closed ileal loop and immunohistochemistry assays were performed as previously described with slight modifications to validate the M-cell targeting property of LL-plSAM-FAdE and the SAM-FAdE protein (Guo et al. 2019; Li et al. 2015). Briefly, SPF BALB/c mice were sacrificed, and the closed ileal loops were prepared. One hundred microliters of LL-plSAM-FAdE, SAM-FAdE (100 μg/mL), or CFAdE (100 μg/mL) was infused into the ileal loop. After incubation for 1 h, the ileal loops were excised and washed with ice-cold PBS, and then fixed and freeze sectioned. The sections of the samples were stained by using rabbit anti-FAdE antibody and Alexa Fluor 647 goat anti-rabbit IgG secondary antibody. Anti-Gp2 monoclonal antibody Alexa Fluor 488 was used to detect M cells in Peyer’s patch. Nuclei were stained using DAPI (Sigma, USA).

### Oral immunization and infection

The outline of prophylactic immunization is shown in Fig. S1a. The SPF BALB/c mice were randomly divided into 4 groups (*n* = 10): the SAM-FAdE protein group, the SAM protein group, the LL-plSAM-FAdE group, and the LL-plSAM group. The LL-plSAM-FAdE and LL-plSAM groups were immunized orally with 3 × 10^9^ CFU of LL-plSAM-FAdE or LL-plSAM, respectively, on days 1, 2, 8, 9, 15, 16, 22, and 23. While, the SAM-FAdE protein group and the SAM protein group were immunized orally with the mixture composed of 100 μg purified SAM-FAdE or SAM protein and 500 μL polysaccharide adjuvant (PA) containing lycium barbarum polysaccharide (20 μg/mL) and chitosan (1%, w/w) on days 1, 8, 15, and 22, respectively. Serum samples were taken at 31 days by tail snips and stored at – 20 ℃. One week after the last oral immunization, all mice were inoculated with *H. pylori* SS1 dilution (0.3 mL, 1 × 10^10^ CFU/mL) by gavage at 31, 33, and 35 days. The mice of different groups were all sacrificed by cervical dislocation after 2 weeks (Nyström et al. 2006).

The outline of therapeutic immunization is shown in Fig. S1b. On days 1, 3, 5, and 7, the SPF BALB/c mice were treated with *H. pylori* SS1 by oral gavage, respectively. One week after the mice infected by *H. pylori*, two mice were killed to make sure the BALB/c mice were successfully infected, then randomly divided into four groups (*n* = 10). On days 15, 16, 22, 23, 29, and 30, two groups were orally with recombinant *L. lactis* LL-plSAM-FAdE (3 × 10^9^ CFU) or the control *L. lactis* LL-plSAM (3 × 10^9^ CFU), respectively. Two additional groups of *H. pylori*-infected mice were orally immunized with the mixture composed of SAM-FAdE or SAM (100 μg) and PA adjuvant (500 μL) on days 15, 22, 29, and 36, respectively. On day 50, the mice were sacrificed by cervical dislocation.

### Assessment of *H. pylori* colonization

The gastric tissue of mice is divided into three parts, after all the mice were killed. Bacterial counts were expressed as colony-forming units (CFU) per gram of gastric tissue (Raghavan et al. 2002). In short, one strip of gastric tissue was homogenized in 0.5 mL of PBS using a tissue homogenizer (The 3rd Gen. TGrinder User’s, TIANGEN, China) after being gently cleaned with PBS to remove any loose stomach contents and weighed. After diluting it in the following ratios: 1:10, 1:100, and 1:1000, 100 μL was plated onto BHI blood plates (Qingdao Hope Bio-Technology Co., Ltd.). These plates were then cultured for 5 days at 37 °C under microaerophilic conditions prior to a colony count being conducted. The following calculation formula was used to convert the *H. pylori* colony counts that were found to be positive into colony-forming units per gram of stomach tissue (CFU/g): *H. pylori* colonization density was equal to bacteria colony count × dilution/gastric weight. Moreover, a real-time quantitative PCR was performed to measure the *H. pylori* SSA gene, and the level of stomach GAPDH expression was measured for normalization as previously described (Moss et al. 2011). One strip of gastric tissue was immediately placed in the urease substrate containing phenol red indicator and kept at 37 ℃ for 4 h and measure OD_550_.

### Gastric histology

For assessment of gastric histology, the gastric tissue strip was embedded in paraffin. About 4-μm-thick sections were stained with hematoxylin and eosin (HE). Then gastritis was graded as described before (Guo et al. 2012). Moreover, immunohistochemical (IHC) analysis was performed to detect *H. pylori* by anti-*H. pylori* antibody (Linc-Bio, Shanghai, China).

### Specific antibodies after oral immunization

The specific serum IgG and secretory IgA (sIgA) antibodies were measured by ELISA. The 96-well ELISA plates were precoated with *H. pylori* lysates (0.5 μg/well). The ELISA signals were then developed by IgA and IgG (Jackson ImmunoResearch, USA).

### Specific T lymphocytes and cytokines

Mouse spleen lymphocytes were isolated and cultured with *H. pylori* lysates (5 μg/mL) for 72 h. After stimulation, CCK-8 assay were performed to analyze the lymphocyte proliferation. Moreover, the supernatant of splenocyte culture was separated and tested for the quantification of cytokines IL-4, IL-17, and IFN-γ by ELISA kits.

### Statistical analysis

The data analysis was performed by GraphPad Prism 5. All data measured during this study were displayed as mean ± standard deviation (SD). Statistical significance was analyzed by Student’s *t*-test. *p* < 0.05 was used as cut off for statistically significant (*p < 0.05, **p < 0.01, ***p < 0.001; ns, not significant).

## Results

### The plSAM vector design and LL-plSAM-FAdE vaccine preparation

The core component SAM DNA sequence is synthesized, which contains signal peptide sequence (Spusp45), leading peptide (Ps), anchoring protein (cA), multiple clone site (MSC), and M cell-targeting peptide (Mtp). Then, SAM was inserted into *L. lactis* plasmid pNZ8148 using the restriction enzymes *Nco* I and *Hin*d III to successfully prepare the plSAM vector (Fig. S2A). An 887bp gene fragment consistent with the theoretical size of SAM was generated (Fig. S2B). Results from gene sequencing confirmed that the gene sequence of the vector plSAM is completely correct (data not shown). And *L. lactis* recombinant plasmid plSAM-FAdE was constructed by inserting the FAdE gene into the plSAM vector (Fig. S2C). The plasmid plSAM-FAdE was verified by gene sequencing (data not shown) and restrictive enzyme digestion (Fig. S2D). Finally, LL-plSAM-FAdE was successfully obtained by transferring the plasmid plSAM-FAdE into *L. lactis* NZ9000.

### Expression analysis of LL-plSAM-FAdE vaccine

**Fig. 1:**
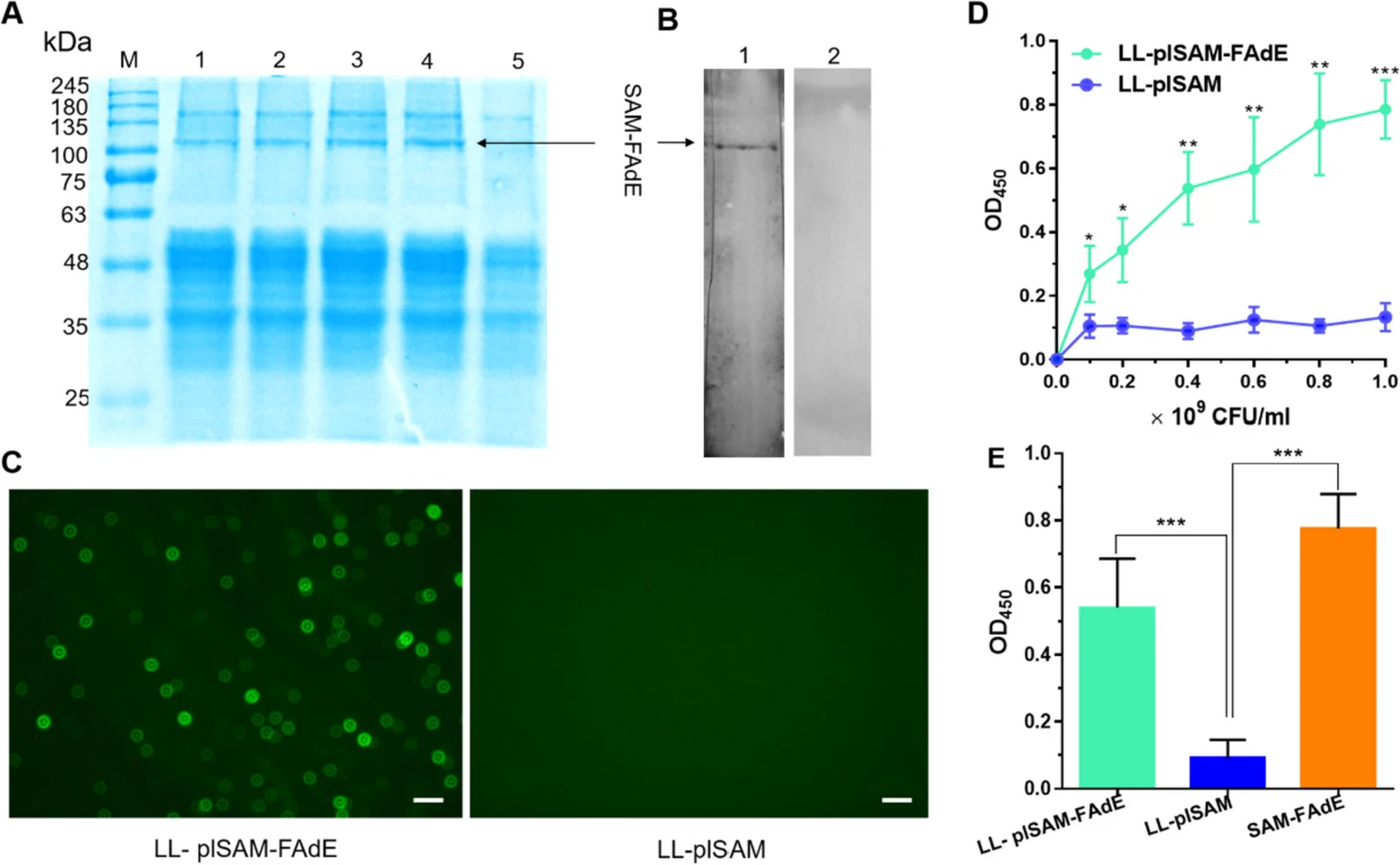
Expression analysis of SAM-FAdE protein in *L. lactis* LL-plSAM-FAdE. A SDS-PAGE analysis. M, protein marker; lanes 1–4, supernatant samples of LL-plSAM-FAdE induced by nisin; lane 5, supernatant samples of LL-plSAM-FAdE without induction. B Western blotting analysis. Lane 1, a mouse anti-FAdE antibody was used to detect the SAM-FAdE protein. Lane 2, serum from a normal mouse was used as a negative control. C Immunofluorescence staining. The bacterial LL-plSAM-FAdE or LL-plSAM were stained FAdE antibody. D Surface display analysis by whole cell ELISA. Defined amounts of bacteria, as indicated on the x-axis, were immobilized in ELISA microplate wells. The SAM-FAdE proteins were detected with primary and secondary antibodies (mouse anti-FAdE and HRP-labeled goat anti-mouse IgG). E Surface display analysis by ELISA. Plates were coated with LL-plSAM-FAdE (5 × 10^8^ CFUs/well), LL-plSAM (5 × 10^8^ CFUs/well), and the SAM-FAdE protein (0.5 μg/well). *: p < 0.05, **: p < 0.01, ***: p < 0.001

LL-plSAM-FAdE was induced with nisin. The cell lysates were prepared and analyzed by SDS-PAGE. An approximately 100.5 kDa fusion protein band in LL-plSAM-FAdE was produced (Fig. 1A). In addition, mouse anti-FAdE polyclonal antibody could recognize the SAM-FAdE protein in LL-plSAM-FAdE as proved by Western blot analysis. However, normal mouse serum did not react with the SAM-FAdE protein (Fig. 1B). Moreover, immunofluorescence analysis confirmed that LL-plSAM-FAdE emitted green fluorescence using mouse anti-FAdE antibody, while no fluorescence was detected in LL-plSAM (Fig. 1C). All above data suggested that SAM-FAdE protein was successfully expressed by LL-plSAM-FAdE. Moreover, the surface display of SAM-FAdE proteins on LL-plSAM-FAdE was confirmed by ELISA (Fig. 1D and 1E), using mouse anti-FAdE polyclonal antibody. A significant increase of SAM-FAdE was found on the surface of LL-plSAM-FAdE, in comparison to that on the negative control (LL-plSAM).

The vaccine antigen protein SAM-FAdE was also produced by the *Escherichia coli* expression system (Fig. s3A) and purified using Ni^2+^-NTA affinity chromatography (Fig. s3B). The SAM-FAdE immunoreactivity was then identified. The result of Western blot suggested that SAM-FAdE protein can be recognized by sera of LL-plSAM-FAdE immunized mice (Fig. s3C). Similarly, the ELISA results confirmed that the sera of *H. pylori*-infected patients were also bound with the SAM-FAdE protein (Fig. s3D).

### LL-plSAM-FAdE M cell-targeting properties and antibody specific assay

**Fig. 2:**
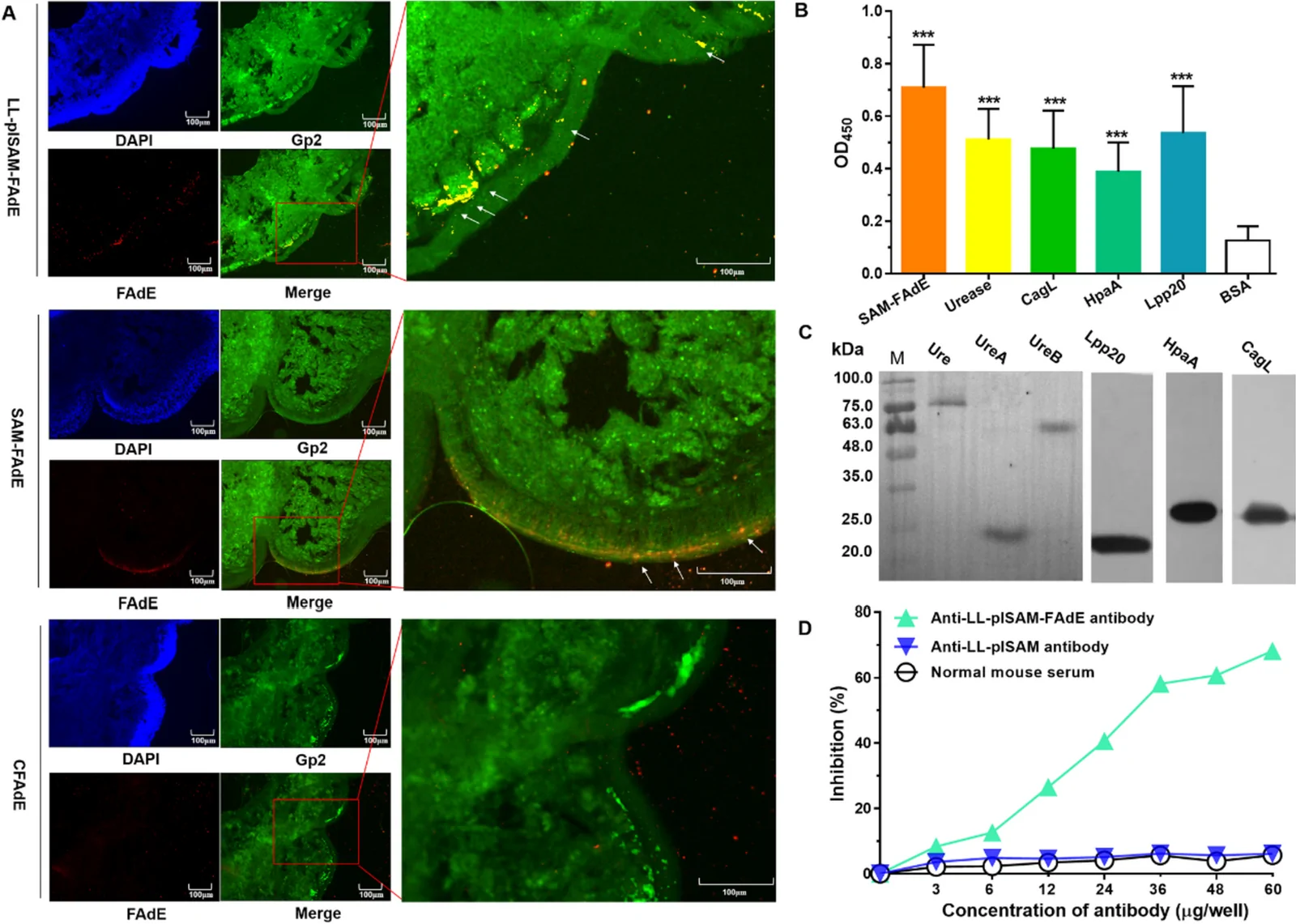
M cell-targeting capacities and antibody specific analysis for LL-plSAM-FAdE. A LL-plSAM-FAdE, SAM-FAdE, or CFAdE were injected into the closed ileal loop. After 1-h incubation the closed ileal loop was excised, washed, fixed, and freeze sectioned. Co-localization of SAM-FAdE binding with M cells was marked by white arrows. B Analysis of antibodies specific for LL-plSAM-FAdE by ELISA. The antiserum was prepared by oral immunization of mice with LL-plSAM-FAdE. ELISA plates were coated with 0.5 μg/well of SAM-FAdE, urease, CagL, HpaA, or Lpp20. BSA was used as a control. The LL-plSAM-FAdE antiserum was diluted at 1:500. C Analysis of antibodies specific for LL-plSAM-FAdE by Western blot. LL-plSAM-FAdE-specific antiserum can react with urease (88.2 KDa), UreA (26.5 KDa), UreB (61.7 KDa), Lpp20 (16.9 KDa), HpaA (26.2 KDa), and CagL (24.6 KDa). D After H. pylori urease incubated with IgG antibodies against LL-plSAM-FAdE, IgG antibodies against LL-plSAM, or normal mouse serum, the urease neutralization assay was analyzed with phenol red indicator and expressed as the inhibition percentage of urease activity

The M cell-targeting property was analyzed by closed ileal loop and immunohistochemistry assays. After the ileal loop injected with LL-plSAM-FAdE, SAM-FAdE, or CFAdE, respectively, FAdE fluorescent signals were recorded. Moreover, M cells in Peyer’s patch were located by M-cell-specific antibody (anti-Gp2-FITC). As shown in Fig. 2A, LL-plSAM-FAdE- or SAM-FAdE-treated mice produced more robust yellow fluorescent signal in Peyer’s patch compared with CFAdE-treated mice, indicating that LL-plSAM-FAdE and SAM-FAdE by conjugating Mtp peptide at it is the C-terminus-enhanced M-cell-targeting property.

The antiserum was prepared by oral immunization of mice with LL-plSAM-FAdE. Experimental results from ELISA (Fig. 2B) and Western blot (Fig. 2C) revealed that LL-plSAM-FAdE owned antiserum specific for the four *H. pylori* adhesins (urease, Lpp20, HpaA, and CagL) and the purified protein SAM-FAdE. However, control BSA protein showed no reaction. Results of urease activity neutralization assay confirmed that antibodies from LL-plSAM-FAdE-immunized mice could effectively inhibit *H. pylori* urease activity. However, antibodies from LL-plSAM immunized mice or normal mouse serum failed to inhibit urease activity (Fig. 2D). Moreover, antibodies obtained from LL-plSAM-FAdE-immunized mice could effectively prevent *H. pylori* adhesion to gastric mucosal cells, but not those antibodies obtained from LL-plSAM-immunized mice (Fig. s4).

### Prophylactic effect of LL-plSAM-FAdE vaccine

**Fig. 3:**
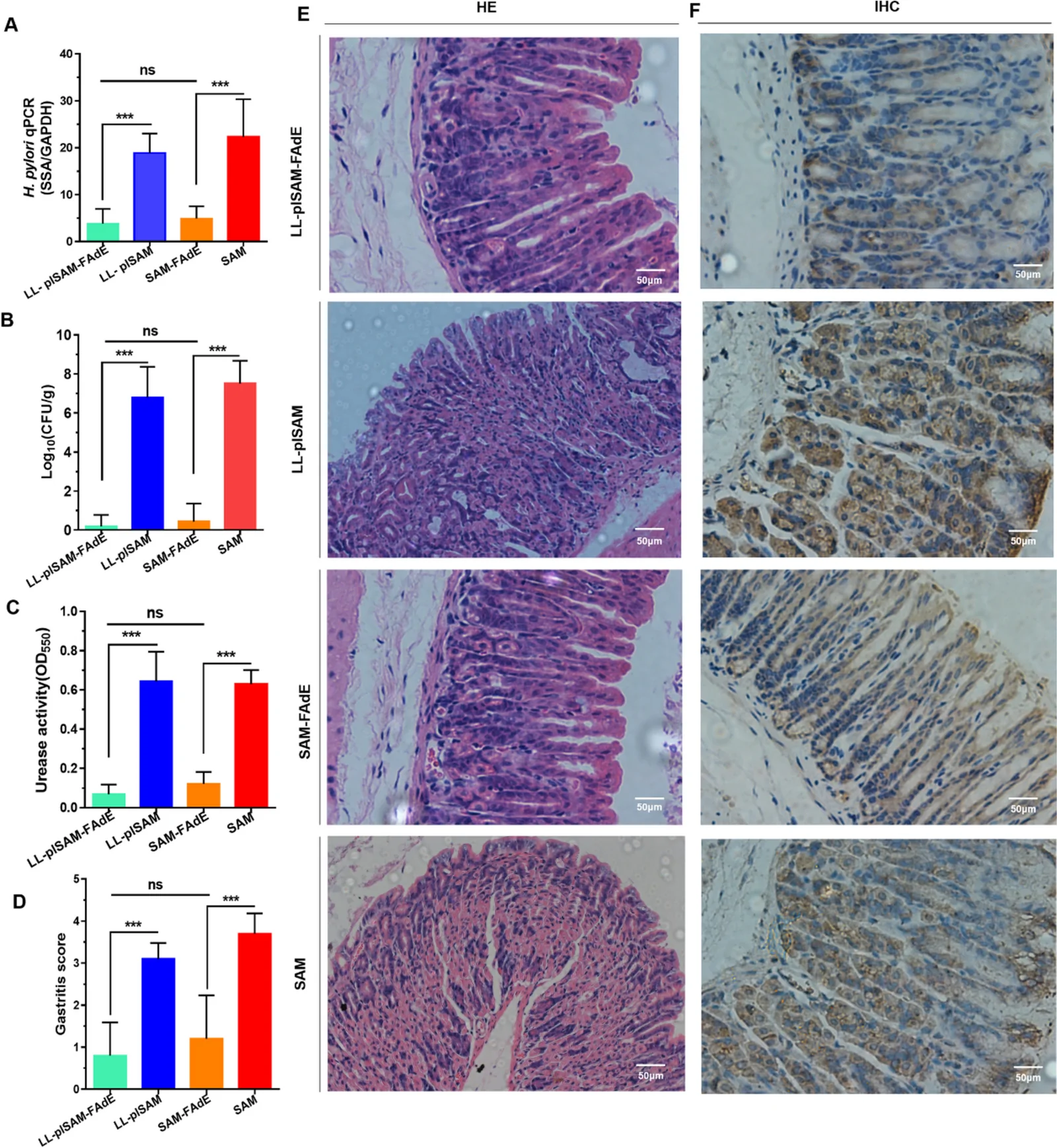
Evaluation of prophylactic effect of LL-plSAM-FAdE. After oral immunization with LL-plSAM-FAdE, LL-plSAM, SAM-FAdE plus PA, or SAM plus PA, the mice were infected with *H. pylori* for analysis. A Q-PCR analysis. B Bacterial quantitative culture. C Urease activity test. D Gastritis grading of gastric tissue showed significantly low inflammatory scores in mice immunized with LL-plSAM-FAdE or SAM-FAdE plus PA compared with mice immunized with LL-plSAM or SAM plus PA. E Representative histopathology images showed mild inflammatory infiltrate of mice orally immunized with LL-plSAM-FAdE or SAM-FAdE plus PA compared with those immunized with LL-plSAM or SAM plus PA (× 100). F Immunohistochemistry (IHC) analysis showed the LL-plSAM or SAM plus PA immunized mice showed positively stained *H. pylori* within a glandular lumen. (***: p < 0.001, ns not significant)

The inhibition of LL-plSAM-FAdE vaccine against *H. pylori* and the prophylactic effect of LL-plSAM-FAdE were examined by quantitative culture of bacteria, Q-PCR, urease activity test, and pathological analysis of the stomach. The results from RT-qPCR (Fig. 3A), quantitative culture of bacteria (Fig. 3B), and urease activity test (Fig. 3C) showed that compared with LL-plSAM, LL-plSAM-FAdE oral immunization significantly decreased the bacterial load and reduced urease activity. More importantly, nine out of 10 mice immunized with LL-plSAM-FAdE did not find *H. pylori* load in the stomach. Gastric inflammatory responses were analyzed by pathological analysis. Representative histological features of gastric mucosa are shown in Fig. 3E, and the gastritis scores of LL-plSAM-FAdE immunized mice were significantly lower than those of immunized mice (Fig. 3D). Moreover, representative IHC images are shown in Fig. 3F, and the results of IHC analysis basically confirmed the histopathologic observations. In addition, oral immunization with SAM-FAdE plus PA can also achieve a good prophylactic effect similar to that of LL-plSAM-FAdE.

### Antibody and cellular immune responses

**Fig. 4:**
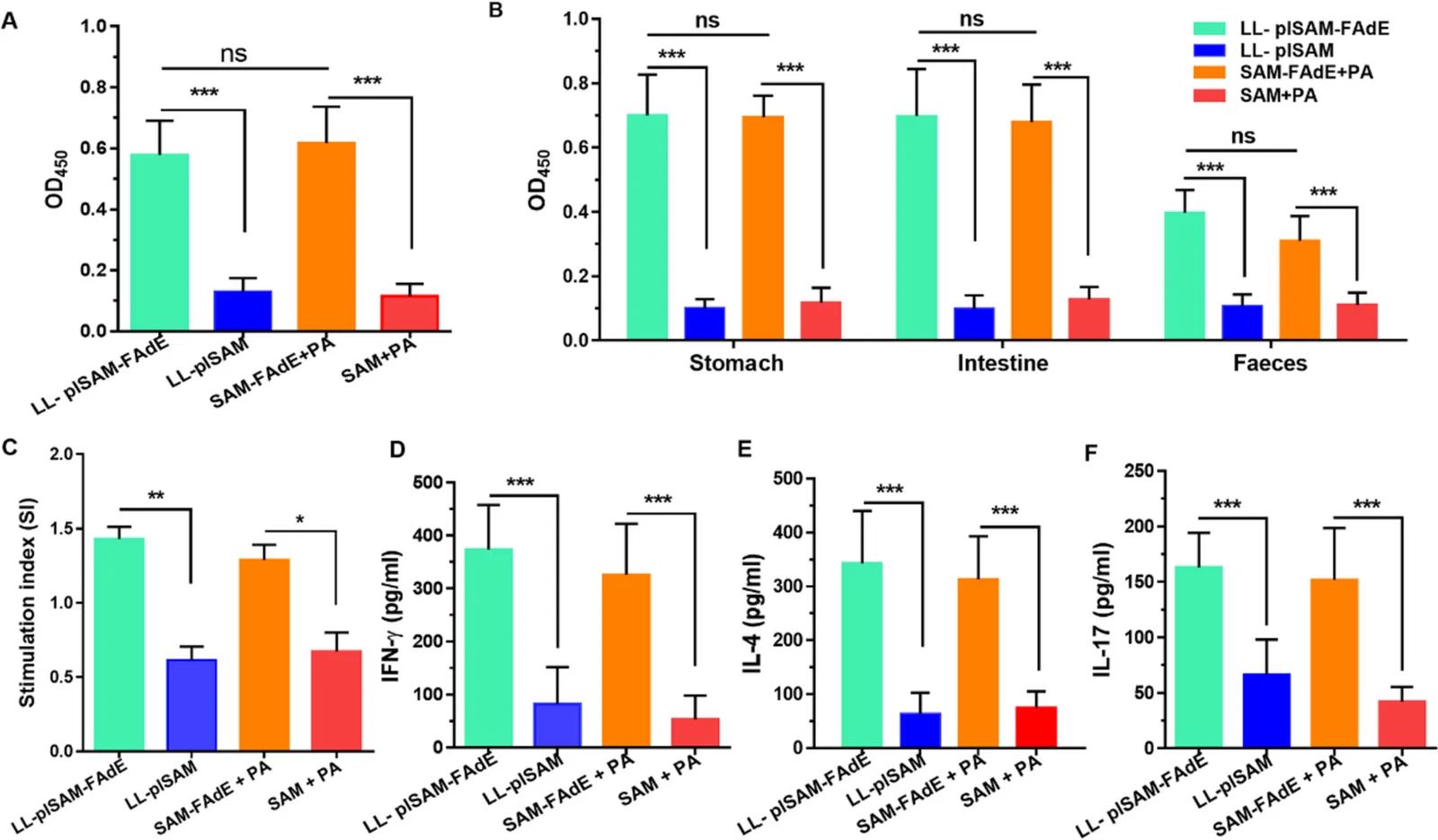
The antibody and cellular immune responses of LL-plSAM-FAdE prophylactic immunized mice. A ELISA analysis of anti- *H. pylori* serum IgG antibodies (1:1000 diluted). B sIgA antibodies of the stomach, intestine and fecal samples. C After immunization and *H. pylori* lysate (5 μg/ml) incubation, splenic lymphocytes were separated and the proliferation rate was determined. Statistical charts of the levels of IFN-γ, IL-4, and IL-17 in the supernatant of splenic lymphocytes after re-stimulation were measured by ELISA (D–F) (***: p < 0.001, ns not significant)

After prophylactically immunized with LL-plSAM-FAdE, the mouse serum IgG and secretory IgA (sIgA) antibodies in gastrointestinal tract and feces of mice against *H. pylori* were examined with ELISA. As shown in Fig. 4A and 4B, after immunized with LL-plSAM-FAdE or SAM-FAdE plus polysaccharide adjuvant (PA), anti-*H. pylori* serum IgG antibodies and sIgA antibodies in the gastrointestinal tract were all increased compared with control mice. These data indicated that LL-plSAM-FAdE or SAM-FAdE plus PA oral immunization could induce significant local and systemic humoral immune responses. ELISA analysis showed that splenic lymphocytes were stimulated with *H. pylori* lysates, and cytokines were presented in the cell culture supernatants. Lymphocytes from LL-plSAM-FAdE or SAM-FAdE protein immunized mice showed significant relatively higher proliferation level than lymphocytes from LL-plSAM or the SAM protein-immunized mice (Fig. 4C). Significant levels of IFN-γ (Fig. 4D), IL-4 (Fig. 4E), and IL-17 (Fig. 4F) were present in the supernatants splenic lymphocytes from LL-plSAM-FadE- or SAM-FAdE-immunized mice compared with those from the control group LL-plSAM or SAM.

### Therapeutic effect of LL-plSAM-FAdE vaccine to eradicate *H. pylori* colonized in the stomach

**Fig. 5:**
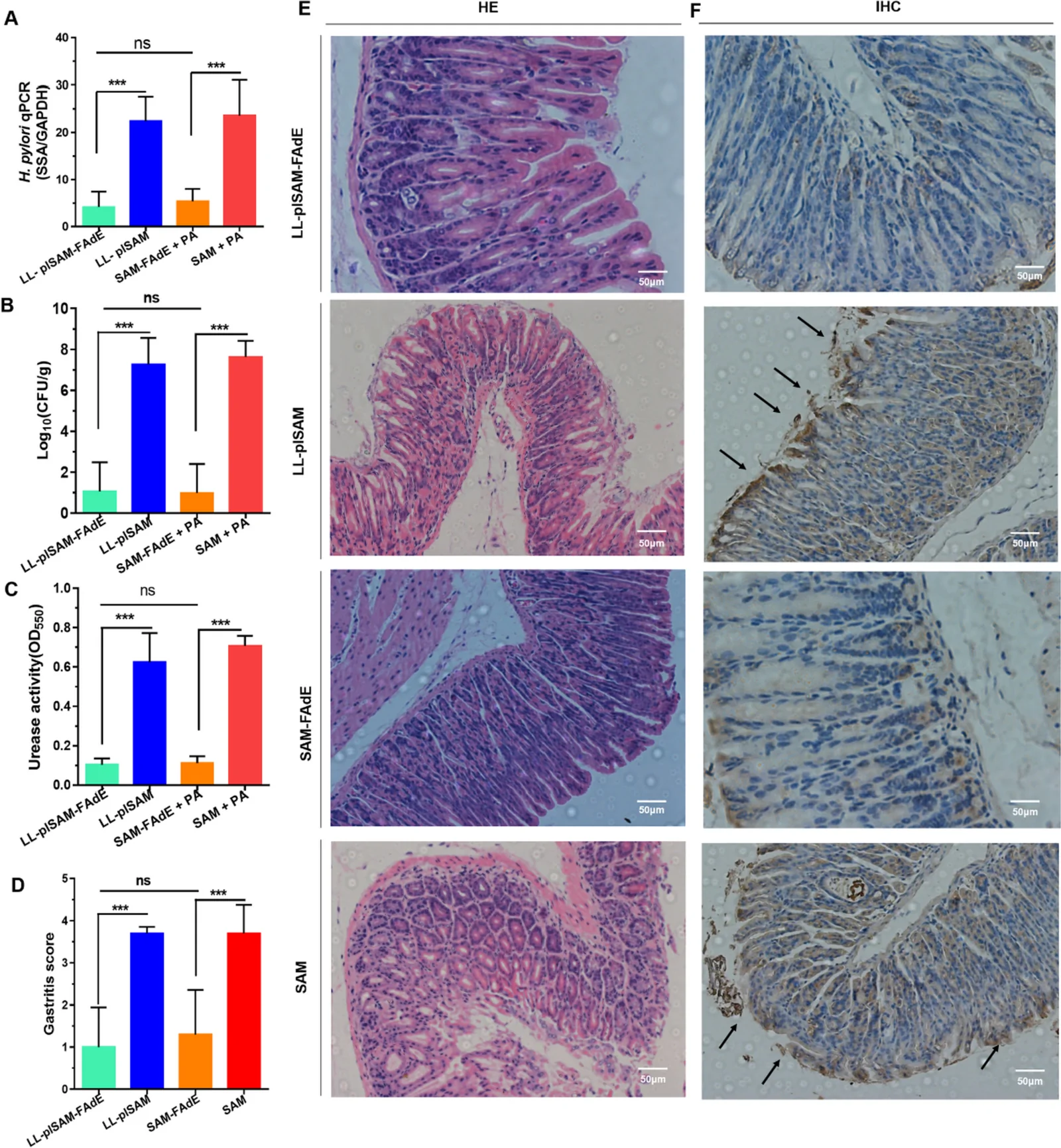
Evaluation of therapeutically effect of LL-plSAM-FAdE. The mice were orally immunized with different vaccines (LL-plSAM-FAdE, LL-plSAM, SAM-FAdE plus PA, or SAM plus PA), and then infected with *H. pylori*. A Q-PCR analysis. B Bacterial quantitative culture. C Rapid urease activity test. D Gastritis grading of gastric tissue. The inflammatory scores of mice immunized with LL-plSAM-FAdE or SAM-FAdE plus PA are significantly lower than those immunized with LL-plSAM or SAM plus PA. E Representative histopathology images (× 100). Oral immunization with LL-plSAM-FAdE or SAM-FAdE plus PA showed mild inflammatory infiltrate in comparison with LL-plSAM or SAM plus PA. F Immunohistochemistry (IHC) analysis (× 100). Mice that were immunized with LL-plSAM or SAM plus PA showed positively stained *H. pylori* within a glandular lumen. (***: p < 0.001; ns, not significant)

The LL-plSAM-FAdE therapeutic effect was examined by immune-treating *H. pylori*-infected mice. All Q-PCR (Fig. 5A), quantitative culture of bacteria (Fig. 5B) and rapid urease activity assay (Fig. 5C) results confirmed that mice orally immunized with LL-plSAM-FAdE or SAM-FAdE plus PA adjuvant showed a remarkable reduction in *H. pylori* colonization number and gastric urease activity. The results of the gastric histopathology experiments confirmed that LL-plSAM or SAM plus PA adjuvant immunized mice had massive both gastric mucosa and gastric submucosa leukocyte infiltration illustrating marked gastric inflammation. However, gastric inflammation was significantly reduced in both LL-plSAM-FAdE and SAM-FAdE plus PA adjuvant immunized mice (Fig. 5D and E). However, the result of the HE staining is only a rough identification of changes in cell morphology and the injury of gastric mucous tissue, and immunohistochemical staining analysis using anti-*H. pylori* to mark the effect of recombinant *L. lactis* LL-plSAM-FAdE to remove *H. pylori* in gastric tissue. The IHC indicated that immunized with LL-plSAM or SAM plus PA adjuvant had *H. pylori* colonization in the stomach. However, seven out of 10 mice immunized with LL-plSAM-FAdE or SAM-FAdE plus PA adjuvant did not find *H. pylori* colonization in the stomach (Fig. 5F), suggesting LL-plSAM-FAdE vaccine and SAM-FAdE plus PA adjuvant had the ability to partially eradicate *H. pylori* colonized in the stomach. IHC analysis further validated the above results.

### LL-plSAM-FAdE therapeutic immunization stimulated specific antibody and cellular immune responses

**Fig. 6:**
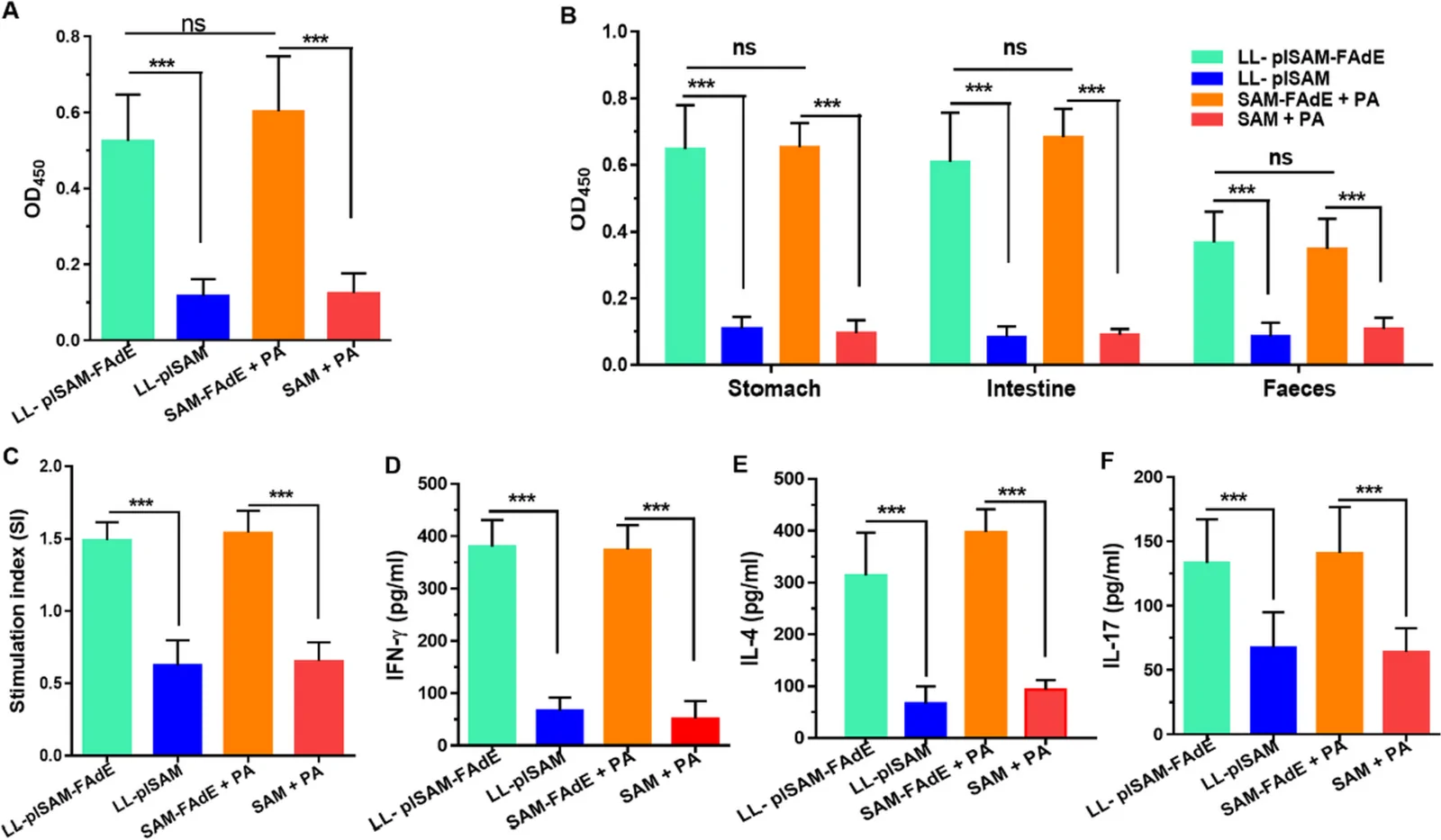
Analysis of antibody and cellular immune responses in *H. pylori*-infected mice after therapeutic immunization with LL-plSAM-FAdE vaccine. A Detection of serum specific IgG antibodies against *H. pylori*. B Detection of mucosal-specific sIgA antibodies against *H. pylori*. C Mouse spleen lymphocyte proliferation assay. After stimulation with *H. pylori* lysates (5 μg/ml), the proliferation of splenic mouse lymphocytes was analyzed. After *H. pylori* lysate stimulation, cytokines (IL-4, IL-17, and IFN-γ) in the culture supernatant of splenic lymphocytes were analyzed (D–F). (*: p < 0.05, **: p < 0.01, ***: p < 0.001; ns, not significant)

The *H. pylori*-infected mice were immunized with the LL-plSAM-FAdE vaccine. Then samples from mouse serum, gastrointestinal mucosa, and feces were examined by indirect ELISA. The results from ELISA showed that after immunized with SAM-FAdE or LL-plSAM-FAdE plus PA adjuvant, the mice could produce *H. pylori*-specific serum IgG (Fig. 6A) and mucosal sIgA (Fig. 6B). However, the mice immunized with LL-plSAM or SAM plus PA adjuvant did not produce any *H. pylori*-specific IgG or sIgA. Mouse spleen lymphocyte proliferation assay was used to further assess the immune function of different vaccines. Results confirmed that, after *H. pylori* lysate stimulation, spleen lymphocytes of mice immunized with LL-plSAM-FAdE or SAM-FAdE plus PA adjuvant showed a significant level of proliferative responses, while spleen lymphocytes of mice provided with LL-plSAM or SAM plus PA adjuvant by oral did not increase significant proliferation (Fig. 6C). In addition, the cytokines in the culture supernatant of mouse spleen lymphocytes were also analyzed by ELISA. Results found that splenic lymphocytes of mice pre-immunized with LL-plSAM-FAdE or SAM-FAdE plus PA adjuvant showed a notable increase in IL-4 (Fig. 6E), IFN-γ (Fig. 6D), and IL-17 (Fig. 6F) after stimulation with *H. pylori* lysates.

## Discussion

*H. pylori* utilizes a number of mechanisms to cause human disease. Gastric mucosa adhesion plays a critical role in *H. pylori* colonization and infection. *H. pylori* adhesins are proteins that are on the surface of *H. pylori* that help in attaching to gastric mucosa (Delahay and Rugge 2012; Evans and Evans 2000). And many outer membrane proteins of *H. pylori* functioned as adhesins become ideal vaccine antigens against *H. pylori* infection (Ansari and Yamaoka 2017; Banga Ndzouboukou et al. 2021). The urease necessary for colonization on gastric mucosa has been proven to be an effective immunogen that causes a strong immune response. The Lpp20 has been considered a potential vaccine candidate (Li et al. 2016). The *H.* *pylori* outer membrane protein HpaA is important for the adhesion to the gastric mucosa (Carlsohn et al. 2006). The pilus protein CagL as a component of *H. pylori* type IV secretion system can bind with integrin α5β1 (Shukla et al. 2013). We previously constructed an epitope vaccine CFAdE containing CTB and poly-epitope peptide FAdE, which showed high *H. pylori* infection inhibition efficacy (Guo et al. 2017b). In this study, we obtained an M cell-targeting recombinant *L. lactis* LL-plSAM-FAdE against four *H. pylori* adhesins. Our results revealed that LL-plSAM-FAdE could express SAM-FAdE on the surface of bacteria and had relatively good M cell-targeting properties. Moreover, LL-plSAM-FAdE vaccine oral immunization induced specific antibodies for urease, Lpp20, HpaA, and CagL. Additionally, oral immunization also significantly inhibited *H. pylori* colonization. The vaccine protection was proved to be correlated with IgG antibody, sIgA antibody, and the antigen-specific CD4^+^ T cells.

The gastrointestinal tract is an important tissue for bacterial control or viral infection, and it is also a major route of entry for potential pathogens. Thus, oral vaccination appears to be a reasonable and effective vaccination measure. Compared with traditional vaccination, oral vaccination can simultaneously induce both effective systemic immune responses and mucosal immune responses (Aziz et al. 2007). In addition, oral vaccination has many advantages including needle-free delivery, and easy and comfortable administration. However, many complex factors in the gastrointestinal tract, such as the highly acidic environment, digestive enzymes, and physical and biological barriers, constrain oral vaccine development (Lycke 2012). For conquering oral vaccine development obstacles, effort has focused on the development of delivery systems for oral vaccines (Lavelle and O’Hagan 2006). As specialized intestinal epithelial cells, M cells can transport a variety of antigens to the underlying lymphoid tissues by transcytosis. Therefore, utilization of M-cell-targeting ligands becomes a hopeful approach for oral mucosal vaccine development (Islam et al. 2019; Kim et al. 2013). Heretofore multiple M cell-specific targets have been identified. For example, Co1 selected by the phage display library method showed an increased ability of uptake of fused antigen and trigger specific immune responses against fused antigen (Kim et al. 2011). Besides, Cpe17 showed significant affinity for an M-cell target receptor claudin-4, by a surface plasmon resonance assay (Ling et al. 2008). Moreover, CKS9 was also identified by the phage display technique (Yoo et al. 2010). New M cell-specific peptide discovery or design is still a research focus that attracts much attention from scholars. In this study, a M-cell-targeting peptide (Mtp) was designed to enhance phagocytosis and transport of vaccine antigens by M cells. The closed ileal loop and immunohistochemistry results revealed that LL-plSAM-FAdE and SAM-FAdE have relatively high M-cell-targeting property compared with the CFAdE protein owing to the Mtp component in the SAM-FAdE protein.

*Lactic acid* *bacteria* (LAB) belong to the Gram-positive, non-spore-forming, acid tolerant, fastidious, and strictly fermentative bacteria genera. LAB has been used since ancient times for food or dairy product fermentation or preservation (Mathur et al. 2020). Several strains of LAB have been regarded as probiotics for a long history of safe exploitation by humans. *L. lactis* has been widely used in the fermentation of food for its food-grade probiotic microorganism status (Song et al. 2017). The use of *L. lactis* as mucosal delivery vehicles, especially display of vaccine antigens on the surface of *L. lactis*, has gradually become a research interest (Cook et al. 2017)*.* In our recent research, a recombinant *L. lactis* LL-plSAM-FAdE was constructed against the four adhesins of *H. pylori (*urease, Lpp20, HpaA, and CagL). The SAM-FAdE protein could be expressed on the surface of bacteria by the LL-plSAM-FAdE display system. More importantly, LL-plSAM-FAdE oral immunization induced antibodies against all four *H. pylori* adhesins.

It is now clear that CD4^+^ T cells (Th cells) are critical for host protection against *H. pylori* infection (Ermak et al. 1998). Based on the cytokine properties, CD4^+^ T cell subset has been grouped into regulatory T cells (Treg), T helper 1 (Th1), T helper 2 (Th2), and T helper 17 (Th17) cells (Caza and Landas 2015). Th1 cells predominantly produce high levels of IFN-γ and TNF-β; Th2 cells secrete IL-4, whereas Th17 cells secrete IL-17 (Lee and Kim 2017). However, it is still debated which subsets of CD4^+^ T cell exert the predominant function in protection against *H. pylori* infection (Bimczok et al. 2010; Ding et al. 2013; Guo et al. 2017a, 2014; Mohammadi et al. 1997; Velin et al. 2009). Our results demonstrated that oral immunization with LL-plSAM-FAdE played a significant protective role against *H. pylori* infection. Moreover, cytokine testing verified that LL-plSAM-FAdE significantly increased three cytokines (IL-4, IL-17, and IFN-γ). Therefore, a mixed Th cell response was induced by LL-plSAM-FAdE, which may be relevant to *L. lactis* NZ9000 and the SAM-FAdE protein. More importantly, both LL-plSAM-FAdE and SAM-FAdE with PA adjuvant induced similar levels of IL-4, IL-17, and IFN-γ, and showed the same *H. pylori* infection inhibition efficiency.

In summary, an antigen-displaying system plSAM consisting SPusp45, PS, cA, multiple clone site (MCS), and Mtp was built to facilitate multivalent epitope vaccine antigen FAdE against four key *H. pylori* adhesins, thus successfully producing recombinant vaccine LL-plSAM-FAdE. The results showed that LL-plSAM-FAdE could express and display SAM-FAdE protein on the *L. lactis* surface. More importantly, LL-plSAM-FAdE could effectively promote M-cell vaccine antigen phagocytosis and transport to stimulate cellular and antibody immune responses against *H. pylori*. The LL-plSAM-FAdE can effectively prevent *H. pylori* infection and even to some extent eradicate *H. pylori* already present in the gastrointestinal tract. Following studies will be carried out in other animal models to evaluate the protective effect of LL-plSAM-FAdE. In addition, the oral vaccines against human *H. pylori* infection clinical trials are expected to be performed in the future.

## Supplementary Information

Below is the link to the electronic supplementary material.

Supplementary file1 (PDF 265 KB)

## Data Availability

All data generated or analyzed during this study are included in this published article and the supplementary information files.
