# Supplementary material for: An M cell-targeting recombinant L. lactis vaccine against four H. pylori adhesins
Source: Appl Microbiol Biotechnol. 2024 Feb 23;108:231. doi: 10.1007/s00253-024-13070-0 (PMC10891252; doi:10.1007/s00253-024-13070-0)
Supplement: Supplementary file 1 [file 253_2024_13070_MOESM1_ESM.pdf]

Supplementary Material

Applied Microbiology and Biotechnology

An M cell-targeting recombinant *L. lactis* vaccine against four *H. pylori* adhesins.

Furui Zhang[2, #], Tianyi Shi[1, #], Zhen Zhang[3, #], Shue Wang[2], Jing Liu[2], Yonghong Li[5], Xuequan Wang[7, *], Kunmei Liu[1, 4, *], Le Guo[2, 6, 7, *].

[1] Department of Pharmacology, College of Pharmacy, Ningxia Medical University, Yinchuan, 750004, China.
[2] School of Laboratory, Ningxia Medical University, Yinchuan, 750004, China.
[3] Department of Geriatrics and Special Needs Medicine, General Hospital of Ningxia Medical University, Yinchuan, 750004, China.
[4] Ningxia Key Laboratory of Cerebrocranial Diseases, Ningxia Medical University, Yinchuan, 750004, China.
[5] School of Public Health and Management, Ningxia Medical University, Yinchuan, 750004, China.
[6] Ningxia Key Laboratory of Clinical and Pathogenic Microbiology, General Hospital of Ningxia Medical University, Yinchuan, 750004, China
[7] Key Laboratory of Radiation Oncology of Taizhou, Taizhou Hospital of Zhejiang Province affiliated to Wenzhou Medical University, Taizhou, 317000, China.

[#]Furui Zhang, Tianyi Shi and Zhen Zhang contributed equally to this article.
*Correspondence to
Le Guo, Email: guoletian1982@163.com, ORCID:0000-0001-9282-7280
Xuequan Wang, Email: yantaiquan@126.com, ORCID:0000-0002-8251-7622
Kunmei Liu, Email: lkm198507@126.com, ORCID:0000-0002-1018-3336.

Table S1. The sequence of SAM-FAdE

**The whole nucleotide sequences of SAM-FAdE (2748 bp)**	CC<u>ATG</u>GAG (*Nco* I)<br>**(SPusp45-Ps)**<br>ATGAAAAAAAAGATTATCTCAGCTATTTTAATGTCTACAGTGATACTTTCTGCTGCAGC<br>CCCGTTGTCAGGTGTTTACGCTTTAGAAATTTCATCAACATGTGATGCT<u> GCATGC (*Sph* I)</u><br>(**cA**)ACTACTTATACCGTCAAATCTGGTGATACTCTTTGGGGAATCTCACAAAGATATGG<br>AATTAGTGTCGCTCAAATTCAAAGTGCGAATAATCTTAAAAGTACCATTATCTACATTG<br>GTCAAAAACTTGTACTGACAGGTTCAGCTTCTTCTACAAATTCAGGTGGTTCAAACA<br>ATTCCGCAAGCACTACTCCAACCACTTCTGTGACACCTGCAAAACCAACTTCACAAA<br>CAACTGTTAAGGTTAAATCCGGAGATACCCTTTGGGCGCTATCAGTAAAATATAAAAC<br>TAGTATTGCTCAATTGAAAAGTTGGAATCATTTAAGTTCAGATACCATTTATATTGGTC<br>AAAATCTTATTGTTTCACAATCTGCTGCTGCTTCAAATCCTTCGACAGGTTCAGGCTC<br>AACTGCTACCAATAACTCAAACTCGACTTCTTCTAACTCAAATGCCTCAATTCATAAG<br>GTCGTTAAAGGAGATACTCTCTGGGGACTTTCGCAAAAATCTGGCAGCCCAATTGCT<br>TCAATCAAGGCTTGGAATCATTTATCTAGCGATACTATTTTAATTGGTCAGTATCTACG<br>AATAAAA<br><u>GGTACC (*Kpn* I)</u>

	**(FAdE)**GGCATCAAACTCAACTATGTAGAGGCAGTGGCCTTAATTTCCGCCCATATCATG<br>GAAGAAGCGCGTGCAGGGAAGAAAACCGCGGCGGAACTGATGCAGGAAGGCCGCA<br>CCCTGCTGAAGCCTGACGACGTTATGGATGGTGTTGCGTCCATGATCCACGAAGTAG<br>GCATTGAAGCGATGTTCCCGGATGGTAGCGTGGAGCTGATTGATATC (*Eco*R *V*)<br>GGTGGTAACCGTCGCATCTTCGGTTTCAACGCCTTAGTGGACCGTCAGGCgGACAAC<br>GAGAGCAAGAAAATTGCTCTACACCGTGCAAAAGAGCGCGGCTTTCACGGCAAGAA<br>AAGCGTCGAACTGATCGATATTGGGGGTAACCGCCGCATTTTCGGCTTCAACGCGCT<br>GGTAGATCGGCAGGCTGAtAACGAAAGCAAAAAgATTGCTCTGCACCGTGCTAAAGA<br>ACGTGGTTTCCACGGTTCTTGTCACCACTTAGACAAATCGATTAAAGAAGATGTCCAA<br>TTCGCTGATAGCCGTATCCGCCCGCAGACCATCGCGGCGGAGGACACCCTGCACGAC<br>ATGGGCATCTTCAGCATCACCTCCAGCGACAGCCAAGCAATGGGtCGTGTAGGgGAAG<br>TGATCACCCGTACCTGGCAAACTGCGGAtAAAAACAAAAAATGCCACCACCTGGACA<br>AAAGTATTAAGGAGGACGTGCAGTTTGCAGATTCCCGTATCCGTCCGCAAACCATCG<br>CTGCAGAAGATACTCTGCATGACATGGGCATCTTCAGTATTACCTCAAGCGACTCCCA<br>GGCGATGGGCCGTGTTGGcGAAGTGATCACTCGTACATGGCAGACTGCGGACAAAAA<br>TAAAAAGTACGAGAAATATTCAGGTGTTTTTCTGGGTCGCGCAGAGGATCTGATTACC<br>AACAACGACGTGGACTACTCAACGAACCAGGCTACGGCGAAAGCGCGCGCAAATCT<br>CGCAGCTAACCTCGGTTCTAGTTGGCCGCTGTACTCTGACGCAAGCGGtCTGGGcTCC<br>TCTTGGCCGCTGTAtTCCGATGCCTCTGGCCTGGGTTCCTCCTGGCCaCTGTACTCCGA<br>CGCCAGCGGTCTGGAATTC (*Eco*R I)<br>CAGGCGCTGGACGAAAAAATCCTTCTGCTGAAACCAGCCTTCCAGTACTCCGATAAC<br>ATTGCAAAAGAATATGAAAACAAATTCAAAAACCAGACGACTCTGAAGGTTGAGGA<br>AATTCTGCAAAACCAGGGTTACAAGGTTATCAACGTCGACTCTTCAGACAAAGATGA<br>CTTCTCGTTTGCTCAGAAAAAAGAAGGCTATTTGGCAGTCGCAATGATCGGTGAGAT<br>CGTTCTGCGTCCGGACCCGAAACGTACCATCCAGAAAAAATCTGAACCGGGCCTGCT<br>GTTCAGTACAGGCCTGGATAAAATGGAAGGTGTGCTCATCCCGGCCGGCTTCGTTAA<br>AGTGACCATCCTGGAACCGATGTCTGGTGAATCTCTGGACAGCTTCACCATGGATCTT<br>AGTGAACTGGATATC (*Eco*R V) CAGGAAAAGTTCTTAAAGCTT (*Hin*d III)GAAGACATTACGAGCGGCCTGAAACAACTGGATAGCACCTATCAGGAAACGAAC<br>CAGCAAGTTCTGAAAAACCTGGACGAAATTTTTAGCACCACGTCACCGTCGGCTAAC<br>AATGAAATCGGCCAGGAAGATGCGCTGAACATCAAAAAAGCAGCAATCGCACTGCG<br>TGGTGACCTGGCACTGCTGAAAGCTAACTTTGAAGCGAATGAACTGTTTTTCATCTC<br>AGAAGATGTCATCTTCAAAACCTATATGAGCTCTCCGGAACTGCTGCTGACGTACATG<br>AAAATTAACCCGCTGGATCAGAATACCGCGGAACAGCAATGCGGCATCTCCGACAAA<br>GTGCTGGTTCTGTATTGTTCTAGA (*Xba* I)<br>TGTAAATCAACACATCCATTATCATGTTCATTTCATCAATTACCAGCTCGTTCACCATTA<br>CCATCATTAGATGCTGGTCAATATGTTTTAGTTATGAAAGCTAATTCATCATATTCAGGT<br>AATTATCCATATTCAATTTTATTTCAAAAATTT TGA (stop codon)<br>AAGCTT (*Hin*d III)
**The whole amino acid sequences of SAM-FAdE (916**	ME (*Nco* I)<br>(SPusp45-Ps) MKKKIISAILMSTVILSAAAPLSGVYALEISSTCDAAC (*SPh* I)<br>(cA)<br>TYTVKSGDTLWGISQRYGISVAQIQSANNLKSTIIYIGQKLVLTGSASSTNSGGSNNSASTT<br>PTTSVTPAKPTSQTTVKVKSGDTLWALSVKYKTSIAQLKSWNHLSSDTIYIGQNLIVSQS

**aa,100.5kDa)**	AAASNPSTGSGSTATNNSNSTSSNSNASIHKVVKGDTLWGLSQKSGSPIASIKAWNHLSS<br>DTILIGQYLRIKGT (*Kpn* I)<br>GIKLNYVEAVALISAHIMEEARAGKKTAAELMQEGRTLLKPDDVMDGVASMIHEVGIEA<br>MFPDGSVELI DI (*Eco*RV)<br>(FAdE)<br>GGNRRIFGFNALVDRQADNESKKIALHRAKERGFHGKKSVELIDIGGNRRIFGFNALVDR<br>QADNESKKIALHRAKERGFHGSCHHLDKSIKEDVQFADSRIRPQTIAAEDTLHDMGIFSI<br>TSSDSQAMGRVGEVITRTWQTADKNKKCHHLDKSIKEDVQFADSRIRPQTIAAEDTLHD<br>MGIFSITSSDSQAMGRVGEVITRTWQTADKNKKYEKYSGVFLGRAEDLITNNDVDYSTN<br>QATAKARANLAANLGSSWPLYSDASGLGSSWPLYSDASGLGSSWPLYSDASGLEF (*EcoR* I)<br>QALDEKILLLKPAFQYSDNIAKEYENKFKNQTTLKVEEILQNQGYKVINVDSSDKDDFSF<br>AQKKEGYLAVAMIGEIVLRPDPKRTIQKKSEPGLLFSTGLDKMEGVLIPAGFVKVTILEP<br>MSGESLDSFTMDLSELDI(*EcoR*V)QEKFLKL (*Hin*d III)<br>EDITSGLKQLDSTYQETNQQVLKNLDEIFSTTSPSANNEIGQEDALNIKKAAIALRGDLA<br>LLKANFEANELFFISEDVIFKTYMSSPELLLTYMKINPLDQNTAEQQCGISDKVLVLYCSR<br>(*Xba* I) CKSTHPLSCSFHQLPARSPLPSLDAGQYVLVMKANSSYSGNYPYSILFQKF

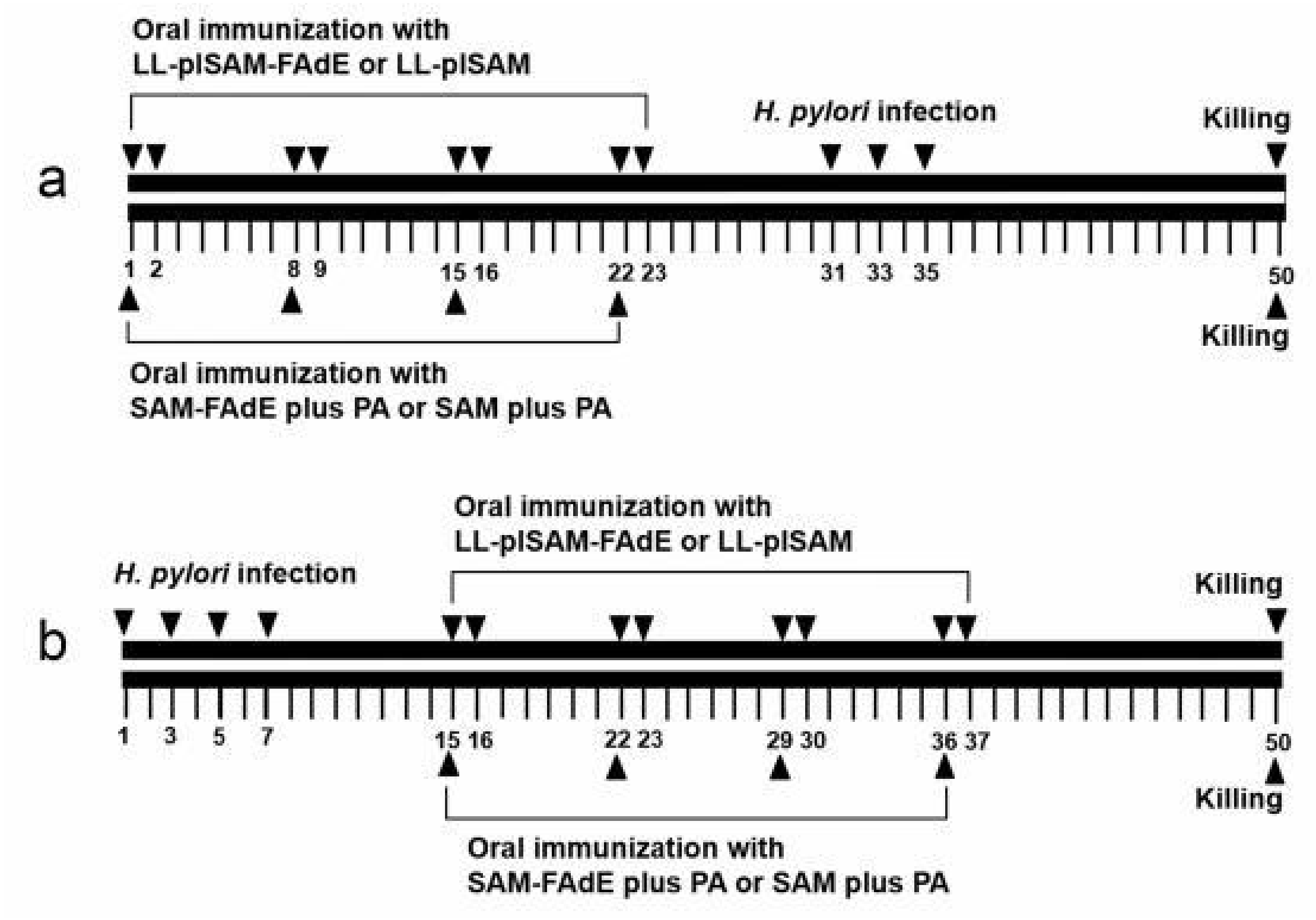

Fig. s1 Flow chart of immunization and infection. (a) For prophylactic immunization, BALB/c mice were randomized into four groups (n = 10). On days 1, 2, 8, 9, 15, 16, 22 and 23, part of mice were orally immunized with LL-plSAM-FAdE or LL-plSAM. While, the others were immunized with the mixture composed of purified SAM-FAdE or SAM protein (100 µg) and PA adjuvant (500 µl) on 1, 8, 15 and 22. One week after the last oral immunization, all mice were treated by gavage with *H. pylori* SS1 at 31, 33 and 35 days. The mice were killed 2 weeks after challenge infections. **(b)** For therapeutic immunization, mice were infected with *H. pylori* by oral gavage on days 1, 3, 5 and 7 respectively. Two groups of *H. pylori*-infected mice were vaccinated with LL-plSAM-FAdE or LL-plSAM orally on days 15, 16, 22, 23, 29 and 30. Two additional groups of *H. pylori*-infected mice were orally immunized with mixture composed of of SAM-FAdE or SAM (100 µg) and PA adjuvant (500 µl) on days 15, 22, 29 and 36. On day 50, the mice were sacrificed.

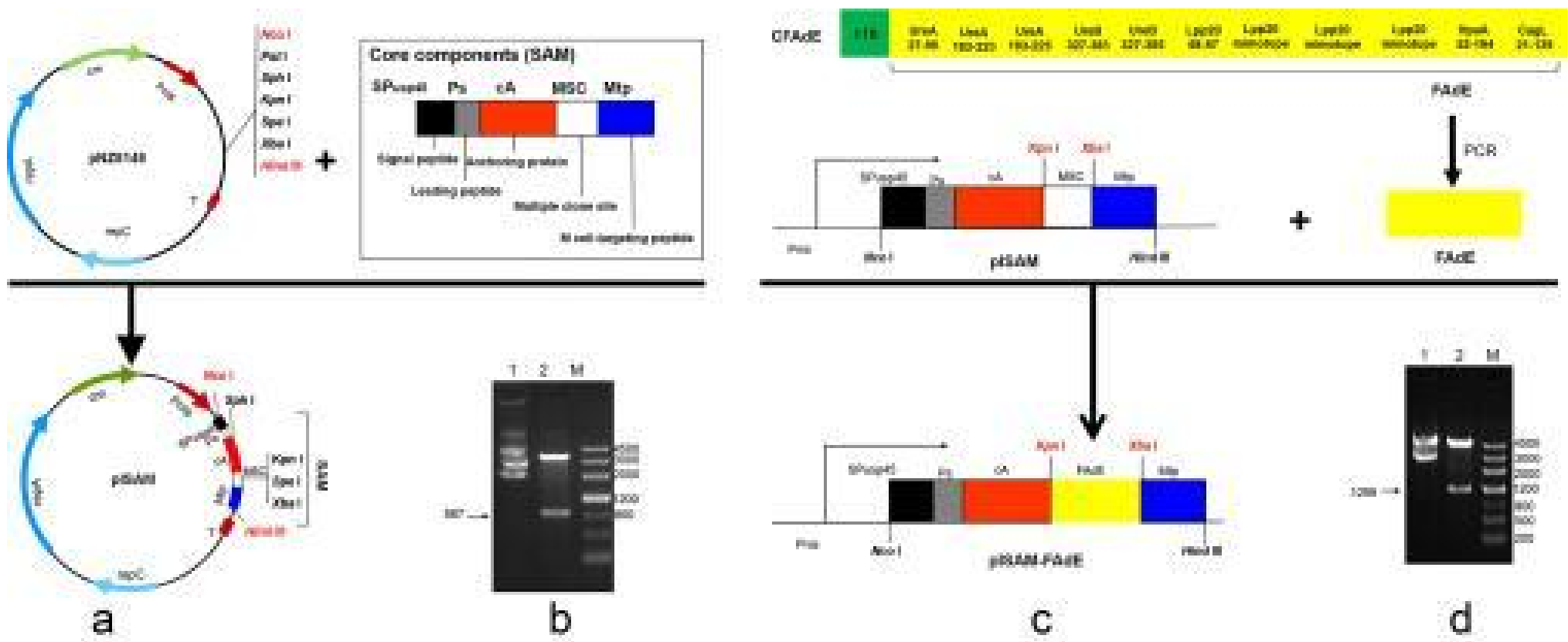

Fig. S2 Design and construction of plSAM and recombinant *L. lactis* vaccine LL-plSAM-FAdE. **(A)** Construction process of plSAM. **(B)** Identification of plSAM by double-enzyme cleavage. **(C)** Construction process of plSAM-FAdE. **(D)** Identification of plSAM-FAdE by restrictive enzyme digestion. The FAdE gene sequence contains two *EcoR* V restriction sites. After restriction enzyme digestion by *Eco*R V, a 1299 bp DNA fragment was produced, which corresponded exactly with the fragment size between the two EcoRV restriction sites in the FAdE gene.

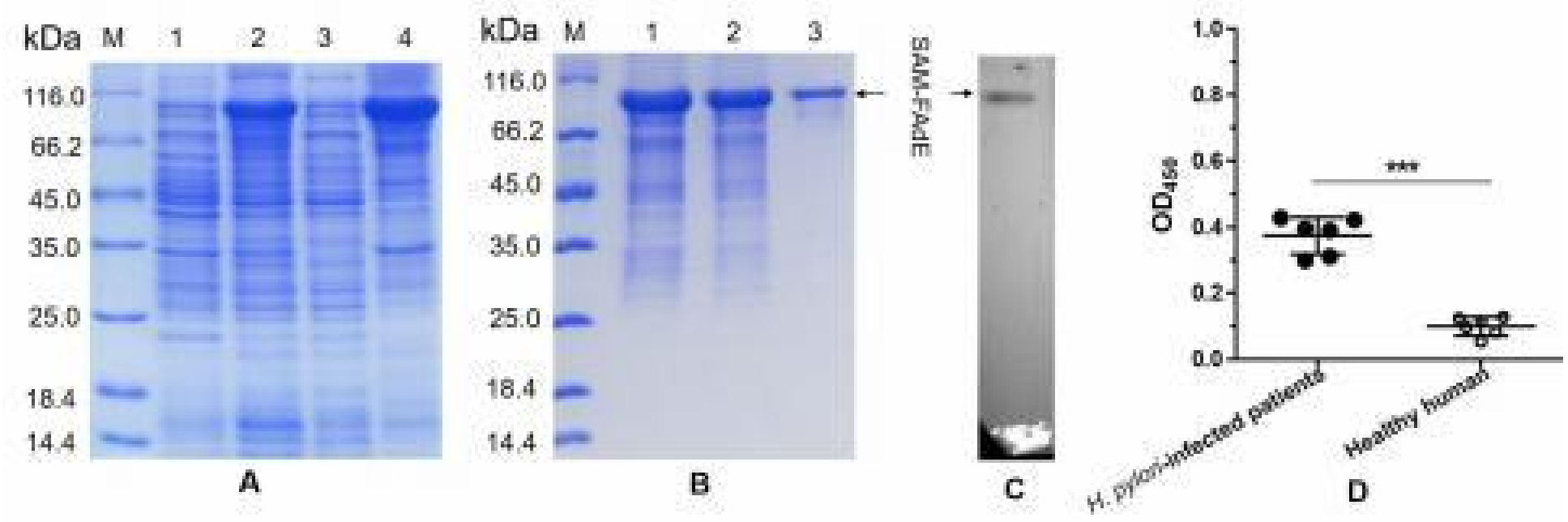

Fig. s3. SAM-FAdE vaccine expression and purification. **(A)** SAM-FAdE were expressed in *E. coli.* Lane M, Protein marker; Lane 1, ArcticExpress (DE3)/peSAM-FAdE without IPTG induction; Lane 2, ArcticExpress (DE3)/peSAM-FAdE with IPTG induction; Lane 3, the soluble proteins of ArcticExpress (DE3)/peSAM-FAdE with IPTG induction; Lane 4, the inclusion bodies of ArcticExpress (DE3)/peSAM-FAdE with IPTG induction. **(B)** Purification of the SAM-FAdE. M, Protein marker; Lanes 1 and 2, the unpurified protein SAM-FAdE; Lane 3, the purified protein SAM-FAdE. **(C)** Western blot. Antiserum specific for LL-plSAM-FAdE also could identified specifically with the SAM-FAdE. **(D)** ELISA. The sera (diluted 100 times) from *H. pylori*-infected patients could identify the SAM-FAdE protein. ***p <0.001.

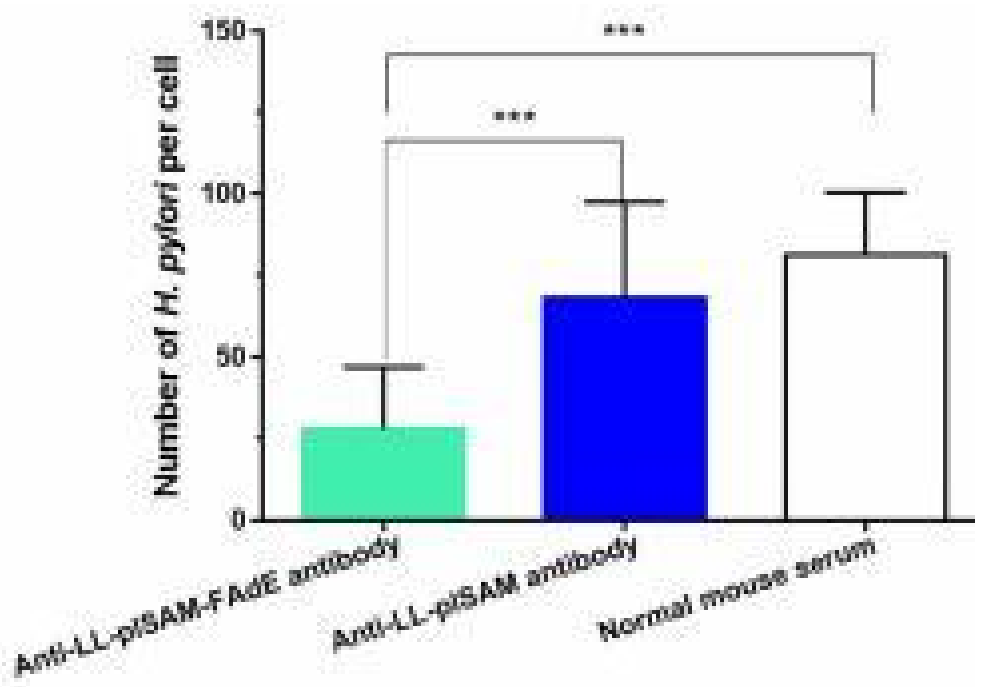

Fig. s4 Inhibition assay of *H. pylori* adhesion to gastric mucosal cells. Gastric mucosal cells (GES-1) were co-cultured with *H. pylori* pretreated with antibodies against LL-plSAM-FAdE, antibodies against LL-plSAM, or normal mice serum. Then, an oil-immersion microscope was used to count the number of *H. pylori* adhesion to GES-1 cells after Kimsa staining. (***: p <0.001).
